# *Epichloë* Fungal Endophytes—From a Biological Curiosity in Wild Grasses to an Essential Component of Resilient High Performing Ryegrass and Fescue Pastures

**DOI:** 10.3390/jof6040322

**Published:** 2020-11-27

**Authors:** John R. Caradus, Linda J. Johnson

**Affiliations:** 1 Grasslanz Technology Ltd., Palmerston North PB11008, New Zealand; 2 AgResearch Ltd., Palmerston North PB11008, New Zealand; Linda.Johnson@agresearch.co.nz

**Keywords:** alkaloids, animal welfare, commercialisation, disease resistance, *Epichloë*, endophyte, microbiology, mutualism, mycology, pest resistance, technology transfer

## Abstract

The relationship between *Epichloë* endophytes found in a wide range of temperate grasses spans the continuum from antagonistic to mutualistic. The diversity of asexual mutualistic types can be characterised by the types of alkaloids they produce in planta. Some of these are responsible for detrimental health and welfare issues of ruminants when consumed, while others protect the host plant from insect pests and pathogens. In many temperate regions they are an essential component of high producing resilient tall fescue and ryegrass swards. This obligate mutualism between fungus and host is a seed-borne technology that has resulted in several commercial products being used with high uptake rates by end-user farmers, particularly in New Zealand and to a lesser extent Australia and USA. However, this has not happened by chance. It has been reliant on multi-disciplinary research teams undertaking excellent science to understand the taxonomic relationships of these endophytes, their life cycle, symbiosis regulation at both the cellular and molecular level, and the impact of secondary metabolites, including an understanding of their mammalian toxicity and bioactivity against insects and pathogens. Additionally, agronomic trials and seed biology studies of these microbes have all contributed to the delivery of robust and efficacious products. The supply chain from science, through seed companies and retailers to the end-user farmer needs to be well resourced providing convincing information on the efficacy and ensuring effective quality control to result in a strong uptake of these *Epichloë* endophyte technologies in pastoral agriculture.

## 1. Introduction

Plants and microbes have long been recognised to co-exist in a symbiotic relationship, and in some cases, they are known to provide benefit to each other in a mutualistic interaction. Some of these microbes have provided technologies that can and have been used as commercial products. This includes rhizobium isolates for improved nitrogen fixation [1], arbuscular mycorrhiza for improved water and nutrient acquisition [2], and *Epichloë* fungal strains for improved animal health and welfare while ensuring grass plant resistance/tolerance to biotic and abiotic stresses [3,4,5,6]. Indeed, there is a view that microbial endophytes have an important role in maintaining productivity levels in environmentally sustainable agricultural systems [7].

Microorganisms are extremely diverse and can exhibit many different biological behaviours related to their symbiotic lifestyle, which allows some of them to function as effective plant protection agents. These differences relate to the type of symbiotic relationship they form with their hosts (mutualistic vs. commensalistic); in planta colonisation patterns (systemic vs. point infections); their level of host-specificity (low vs. high); means of propagation (horizontal vs. vertical); and endophytic lifestyle (obligate vs. facultative) [8]. The symbioses of the *Epichloë* fungal species with host grasses, of the family Pooidae [9,10], can span the continuum from antagonistic to commensal or mutualistic [11], but here, the focus will be largely on the asexual mutualistic types.

Asexual *Epichloë* endophytes exhibit the characteristics of mutualism, systemic infection, high host specificity, vertical (maternal) transfer, and an obligate lifestyle [8] that in many ways make this microbial technology unique and in part explains why as a commercial product, it has been so successful [12]. They are known to produce a large range of secondary metabolites of which the alkaloids are the most well characterised [3,13]. The aim here is to review this mutualistic relationship to determine (1) the origins of *Epichloë* strain variation, (2) reasons for its importance in many temperate grass pastures, (3) methods of managing its negative and positive characteristics, (4) how effective delivery of commercial *Epichloë* technologies has been achieved, and (5) how further research opportunities can continue to add value to this economically important relationship, which underpins sustainable pastoral farming practices in managed temperate grasslands.

## 2. *Epichloë* Endophytes

### 2.1. Epichloë Taxonomy

The *Epichloë* genus contains two major categories of fungal organisms, such that of the 43 documented *Epichloë* taxa associated with grasses (Table 1) [14], 14 are known to develop sexual structures with viable ascospores, while for the other 29 taxa the sexual state has not been observed [15]. Prior to 2014 the *Epichloë* genus contained only the sexual forms (teleomorph), but now also contains the asexual forms (anamorph), which had previously been classified as *Neotyphodium* [15], and prior to that, *Acremonium* [16]. This change resulted from a requirement that a single genus name is to be used for all stages of the development of a fungal species [17].

### 2.2. Epichloë Diversity and Origins

*Epichloë* endophytes have been found in more than 100 grass species, which have evolved in most temperate regions of the world (Table 1) [18,19]. However, it is acknowledged that endophyte infection is rare in grasses endemic to Australasia [20], and sub-Saharan Africa [21] in comparison to the wide range of infection found in the wild, uncultivated grasses of the Northern Hemisphere and in particular Europe [5] and Asia [22]. Indeed, the most important temperate grass species from an economic viewpoint, namely *Lolium* and *Festuca* species, have originated in Europe and North Africa [23]. In South America, most collections of grasses containing *Epichloë* have been made in the Patagonian steppe [24]. While modern cereals are not naturally infected by *Epichloë*, a range of their progenitor species in the genera *Elymus* and *Hordeum* are frequently infected [25]. However, when *Epichloë* strains from the wild grasses have been inoculated into rye (*Secale cereale*), individual genetically distinct host genotypes show morphological phenotypes that range from heavily stunted through to some that resemble healthy uninfected plants [26,27]. *Epichloë* infection has also been achieved into the wheat genome using Chinese spring wheat substitution lines [28].

**Table 1:** Infection of *Epichloë* species in temperate grasses by region – for more extensive and detailed listings [14,15,22,29,30,31,32].

Grass Genus	*Epichloë* Species	Reference
Europe/North Africa		
*Lolium canariense*	*E. typhinum* var. *canariense*	[33]
*Lolium multiflorum*	*E. occultans*	
*Lolium perenne*	*E. hybrida*	[34]
*Lolium rigidum*	*E. occultans*	[15]
*Agropyron repens*	*E. bromicola*	[35]
*Agrostis*	*E. baconii*, *E. amarillans*	[32]
*Anthoxanthum*	*E. typhina*	
*Brachyelytrum*	*E. brahyelytri*	
*Brachypodium*	*E. sylvatica*, *E. typhina*	
*Dactylis glomerata*	*E. typhina*	
*Elymus*	*E. elymi*	
*Festuca arundinacea*	*E. coenophialum*	
*Festuca giganteus*, *Festuca rubra*	*E. festucae*	
*Glyceria*	*E. glyceriae*	
*Holcus*	*E. clarkii*	
*Leymus*, *Bromus*	*E. bromicola*	
*L. perenne*	*E. festucae* var. *lolii*, *E. typhina*, *E. lolii*	
*Phleum*	*E. typhina*	
*Poa*	*E. typhina*	
*Sphenopholis*	*E. amarilians*	
*Festuca pratensis*	*E. uncinatum*	
	*E. siegelii*	[36]
*Hordelymus*	*E. disjuncta*, *E. danica*, *E. hordelymi*, *E. sylvatica* subsp, *pollinensisi,*	[15,37]
*Holcus mollis*	*E. mollis*	[15,38]
**Asia**		
*Achnatherum*	*E. ganusuensis*, *E. sibirica*	[22]
	*E. chisosum; E. inebrians*	[29,39]
	*E. funkii*	[15]
*Brachypodium*, *Bromus*, *Elymus*, *Leymus*	*E. bromicola*	[22]
*Calamagrostis*	*E. stromatolonga*	
*Festuca*	*E. sinofestucae*	
*Elymus*, *Elytrigia*, *Festuca*, *Hordeum*, *Poa*, *Roegneria*, *Stipa*	*E.* spp.	
*Poa*	*E. liyangensis*	[40]
*Roegneria*	*E. sinica*	[22]
	*E. yangzii*	[41]
**North America**		
*Ammophila*	*E. amarillans*	[42]
*Brachyelytrum erectum*	*E. brachyelytri*	[11]
*Bromus laevipes*	*E. cabralii*, *E.* spp.	[43]
*Cinna arundinacea*	*E. schardlii*	[44]
*Elymus*	*E. elymi*	[11]
*Elymus canadensis*	*E. canadensis*	[15,45]
*Festuca arizonica*	*E. huerfanum*, *E. tembladerae*	[29]
*Glyceria striata*	*E. glyceriae*	[11]
*Poa alsodes*	*E. alsodes*	[46]
*Poa secunda* subsp. *junicolia*	*E. poae*	[31]
**South America**		
*Bromus setifolius*	*E. typhina* var. *aonikenhana*	[47]
	*E. typhinum*	[48]
	*E. tembladerae*	[15]
*Bromus auleticus*	*E. pampeana; E. tembladerae*	
*Festuca argentina*, *F. hieronymi. Poa huecu*	*E. tembladerae*	[49]
*Hordeum comosum*	*E. tembladerae*, *E. amarillans*, *E. typhina hybrids*	[24]
*Melica ciliata*	*E. guerinii*	[15]
*Melica decumbens*	*E. melicicola*	[29,50]
*Phleum alpinum*	*E. cabralii*	[47]
	*E. tembladerae*	[15]
*Poa*, *Briza*, *Festuca*, *Melica*, *Phleum*	*E. tembladerae*, *E. pampeana*	[50,51,52]
**Australia**		
*Echinopogon* spp.	*E. australiense*	[50,53]
**New Zealand**		
*Echinopogon ovatus*	*E. aotearoa*	[50]
*Dichelachne micrantha*	*E. australiensis*	[20]
*Poa matthewsii*	*E. novae-zelandiae*	
**Sub-Saharan Africa**		
*Festuca costata*	*E.* spp.	[21]
*Melica* spp.	*E. melicicola*	[50]

*Epichloë* strains have been classified as either hybrid (being the result of a cross between two or more species) [54,55] or non-hybrid. While hybrids have interspecific origins, there is one known exception, *E. schardlii*, which has resulted from intraspecific hybridisation [15,44]. At least half of all known *Epichloë* species are hybrid types [10,15,29,56] and with one rare exception [40], all hybrid species are asexual [48,57]. However, that does not mean that non-hybrid types are necessarily capable of sexual reproduction [37]. Interspecific hybridisation most likely occurs via somatic cell fusion followed by fusion of nuclei [54,56]. *Epichloë* is notable for having more interspecific hybrids than any other fungal genus [34]. Whereas horizontally transmissible species have haploid genomes, producing ascospores [58], most of the strictly seedborne mutualists, such as most *Epichloë* species, are hybrids with heteroploid (aneuploid or polyploid) genomes [29]. Yet even some of these can form epithelial growth that produce conidia [31,59] with the potential to horizontally transmit, but the dominant and more successful form of transmission is still vertical transmission through the host plant seed [10]. Direct infection by germinating conidia has not been documented [60].

At least in some instances, hybridisation came after the strain became seedborne rather than being caused by the seedborne habit suggesting a selective advantage of hybridization for the mutualistic endophytes. Hybrids are likely to contain more genetic variation, which may lead to improved adaptation to biotic and abiotic stresses of their host plants [10,29,30,56,61]. There is also a general hypothesis that interspecific hybridisation provides greater genetic variation and hence, a wider adaptation range in stressful environments than intraspecific hybridisation [56,62]. However, when comparing hybrid and non-hybrid *Epichloë* strains on controlled environments, there is no evidence of niche expansion of *Epichloë* hybrid-infected plants [63]. They also showed that non-hybrid endophytes increased seed production of their hosts, whereas hybrid endophytes reduced it, suggesting a fitness advantage for plants hosting non-hybrid endophytes.

Diversity within the *Epichloë* genus can be characterised by the types of alkaloids they produce in planta [3,64,65]. Four major classes of alkaloids are known to be produced by *Epichloë* strains. These include lolines (saturated 1-aminopyrrolizidines), indole diterpenes (lolitrems, epoxyjanthitrems), ergot alkaloids (main terminal product is ergovaline), and peramine (a pyrrolopyrazine alkaloid) [30,66]. Naturally occurring strains of *Epichloë* may produce from none to all four types of these known alkaloids. Additionally, most of the secondary metabolite pathways that result in producing the known chemistry are complex and have many intermediate compounds, some of which have been shown to have bioactivity [3,67]. There is still a considerable amount of unknown bioactivity associated with *Epichloë* endophytes and conversely, there are known secondary metabolites with undescribed or putative functions. *Epichloë* strains AR48 and AR47, for example, have been shown to control cutworm moth caterpillar (*Agrotis ipsilion*), but the alkaloid associated with that control is as yet unknown [68]. Whereas, examples of the latter are the non-alkaloid compounds epichloecyclins, which are cyclic ribosomally synthesized and post translationally modified peptides (RiPPs) with no known function [69] and a hybrid peptide-polyketide named Dahurelmusin A with only putative insecticidal activity [70]. While it is the endophyte strain that carries the genes required for alkaloid expression it is unknown factors associated with the host genetics [71,72,73], including the expression of plant hormones [74], that moderate alkaloid expression. Alkaloid expression levels can be further modified quantitatively by the environment [75,76,77,78,79,80]. These alkaloids are either not expressed or at very low levels when *Epichloë* is grown in axenic culture, but are highly expressed in planta [81,82,83,84]. The epigenetic regulation of the ergot alkaloids and lolitrems via chromatin remodelling also plays a critical role in the symbiosis-specific expression of these alkaloid pathways [81,85,86].

Distribution of alkaloids can vary within the plant and they are not necessarily correlated with the distribution of fungal hyphae associated with the *Epichloë* endophyte [87]. In perennial ryegrass, lolitrem B accumulates in older tissues, ergovaline is concentrated in the stem and basal leaf sheath of intermediate age tillers, and peramine is evenly distributed across all leaf tissues [88,89]. For flowering ryegrass plants, the seed component contains about 75% of the total peramine present in the plant [90]. In fescue plants, loline can be found in both the shoot and root tissue [91,92]. In shoot tissue, the highest levels of loline occur in the inflorescence, followed by meristem and then pseudostem [93]. The highest peramine concentrations have been found in young leaves of meadow fescue in early spring and in panicles (spikelets, seeds) and leaf pseudostems during the period of vegetative growth in late summer and autumn [94].

### 2.3. Epichloë Mutualism

Mutualism occurs when each participant receives a net benefit from the association [95,96]. *Epichloë* endophytes can form mutualistic symbiotic associations [97,98,99] within the aerial tissues of some temperate cool-season grasses of the subfamily Pooideae [26,55]. Within this subfamily, 50% of the 14 tribes have species that host *Epichloë* [14,55] (Table 1). Discoveries mostly over the last decade, have revealed dynamic and complex cellular and molecular responses critical for establishing and maintaining mutualistic symbiotic interactions (previously reviewed [99]). These include nutrient related processes such as regulation of apoplastic iron homeostasis [100,101,102], epigenetic regulation [81,86], and signalling pathways such as Nox produced reactive oxygen species (ROS) [103], calcineurin signalling [104], lipid signalling [105], G protein and adenosine 3′, 5′ -cyclic monophosphate (cAMP)/cAMP-dependent protein kinase (PKA) signalling [106,107], stress-activated mitogen-activated protein (MAP) kinase pathway [108], and the cell wall integrity (CWI) mitogen-activated protein kinase (MAPK) pathway [109]. Transcriptomic studies indicate that symbiosis establishment requires significant host reprogramming with genes associated with photosynthesis, stress, plant hormone biosynthesis and perception, cell membrane regulation, and plant defence [110,111,112,113,114].

### 2.4. Epichloë Systemic Infection

*Epichloë* systemically infect plant tissues [115,116,117] but are only found in the aerial parts of grass plants. Establishment of infection requires colonisation of the meristematic tissues of the shoot apex, which occurs by extensive hyphal branching [118]. To systemically colonise aerial tissues, hyphae grow between leaf cells and as the leaf extends, hyphae attached to host cell walls commits the hyphae to grow by intercalary expansion (so that hyphal filament length increases as the leaves expand) to avoid breakage in a manner that is highly regulated and synchronised with host leaf expansion [118,119].

### 2.5. Epichloë Host Specificity

The *Epichloë* fungus has co-evolved with it host grass over millennia [120] to the point where the genome of *Epichloë* has genes for improved host compatibility [121]. Moving *Epichloë* strains across grass taxa has been difficult and largely unsuccessful, suggesting that *Epichloë* species and even some strains have developed through co-speciation and are essentially host species specific [41,122]. Strong host specificity of *Epichloë* endophytes is related to both host species and their provenance [123].

### 2.6. Epichloë Vertical Transmission

Vertical transmission of *Epichloë* through host seeds [124] is a critical element that allows the transfer of the endophyte to successive generations through seed production processes and delivery to end user pastoral farmers. It has been hypothesised with good evidence that vertical transmission results in enhanced capability of host protection [30]. The success of vertical transmission can depend on the compatibility of the endophyte strain with the host genetics. In seed produced from natural associations, the fungus can be associated in seed at close to 100% [125], however in Europe where *Epichloë* co-evolved along with ryegrass and tall fescue, rates can be lower [126]. The reduced rate is thought to be due to the endophyte not necessarily being beneficial for the host plants in all environments [127,128] and/or an imperfect spread to all tillers of the plant resulting in the lack of transmission through seed [129], or reduced viability of the endophyte in seed [130,131]. For novel associations created by moving endophyte strains into new host germplasm, the rate of transmission can be much lower [132,133], although it has been possible to use host plant selection to improve the transmission rate, showing the importance of host plant genetics [134] for vertical transmission.

While asexual *Epichloë* endophytes are obligate with no free living form in nature, they are totally reliant on their host plant for survival and can rapidly lose viability when seed is stored at high temperatures and high humidity [135], and over about 6 months if stored at ambient temperatures [136]. To maintain endophyte viability in seed, storage at low temperatures (<5 °C) and low relative humidity (<60%) is recommended [130].

## 3. Impact of *Epichloë* Endophytes in Pastoral Systems

### 3.1. Animal Health and Welfare

*Epichloë* endophytes were primarily discovered as a result of animal health and welfare issues caused by alkaloids resulting from the mutualistic association, namely in tall fescue [137,138] and ryegrass [139]. *Epichloë* in tall fescue was shown to be associated with a condition in the USA known as fescue toxicosis [140], which has been estimated to create production losses of about US$1 billion per year [141]. This was particularly evident in cattle and dairy cows [142,143,144], largely because they were the most commonly used grazing animal in the USA, but it also occurs with sheep [145,146,147], goats [148], horses [149], deer [150], and alpacas [151]. The offending alkaloid causing fescue toxicosis has been identified to be ergovaline [152] which in the rumen, breaks down to lysergic acid [153], but a range of other ergot alkaloids may be implicated [154,155] (Table 2).

For perennial ryegrass the presence of *Epichloë* was associated with ryegrass staggers in New Zealand [140,156,157] caused by the alkaloid lolitrem B [158], although this condition was recorded many years before that [159]. Lolitrem B, a lipophilic compound, is a neurotoxin that affects muscular coordination resulting in tremors [152,158]. It also impacts on respiratory, cardiovascular, and digestive systems [160]. There are many lolitrems that have been characterised and labelled by a letter (A to N) and differ by the presence or absence of an I ring and the number of hydroxyl and aryl substitutions [161]. The tremorgenic properties of these lolitrem compounds can vary considerably (Table 2).

However, for the *Epichloë* association with ryegrass the presence of ergovaline can cause increases in body temperature [162,163] and respiration rate [163,164] of sheep and cattle. Comparisons of sheep grazed on endophyte free and endophyte infected ryegrass showed that the impact of *Epichloë* endophyte was much greater than just causing stagger events [165]. Also evident were reductions in daily liveweight gains and plasma prolactin, and increased presence of daggs, incidence of flystrike, and rectal temperatures (Table 3).

In Australia, the presence of *Epichloë* endophytes in perennial ryegrass causes a condition termed “perennial ryegrass toxicosis”, which has been attributed to the expression of both ergovaline and lolitrem B [156,166]. A severe perennial ryegrass toxicosis epidemic, which occurred in 2002, resulted in an estimated 100,000 sheep deaths.

While much is known about the toxic effects of ergovaline and lolitrem B less is known about the impact of other alkaloids associated with *Epichloë* infection [13]. A summary of known impacts of *Epichloë*-associated alkaloids on animal health and welfare is provided in Table 2. Many alkaloids also accumulate in the seed [88], acting as feeding deterrents for birds and rodents [167].

Lolines [168] and peramine [84,169] alkaloids are considered not toxic to grazing animals (Table 2). Peramine is unique and not known outside of the *Epichloë* genus [82,170]. For meadow fescue and tall fescue it is possible to identify endophyte isolates inducing the production of zero, low, or high loline concentrations, while for perennial ryegrass, endophytes strains have not been found that express loline [171]. Up to seven types of loline have been shown to be expressed by *Epichloë* endophytes in fescues, with N-formylloline (NFL) and N-acetylloline (NAL) being the most abundant [172] and along with N-acetyl norloline (NANL) the most bioactive [173]. There has been a report of loline and, in particular NANL causing equine fescue oedema [174], but further more detailed and thorough work has shown this is not the case and that lolines or NANL are unlikely to be the causative agent of this disease [175]. Lolines are extensively metabolised in the digestive tract of sheep prior to absorption and/or in the liver or other tissues following absorption resulting in low levels of excretion in urine and faeces [176].

**Table 2:** Documented effects of alkaloids expressed by *Epichloë* on animal health and welfare.

Alkaloid	Animal Effect	Action and Qualifying Information	Reference
**Ergot Alkaloids** [177]			
Chanoclavine	No toxic effects at levels found in grasses	May lower prolactin serum levels at high concentrations	[178,179]
Dehydroergovaline	May contribute to toxicity	Present only in fescue	[13]
Ergine	Stupor	High levels in *Stipa robusta* and *Achnatherum inebrians*	[13,180,181]
Ergocornine	Fescue toxicosis	Intermediate in vasoconstriction between ergovaline and lysergic acid	[154]
Ergocristine			
Ergocryptine			
Ergonovine			
Ergonovine	Fescue toxicosis	Lowered skin temperature, heart rate, and prolactin and had a higher respiration rate and blood pressure	[182]
Ergotamine	Fescue toxicosis	Similar vasoconstriction effect as ergovaline	[183]
Ergotamine	Fescue toxicosis	Fever, diarrhoea, weight loss, laboured breathing, salivation, low prolactin	[182,184]
Ergosine			
Agroclavine			
Ergovaline	Fescue toxicosis/fescue foot	Inability to regulate body temperature; vasoconstrictor; regulates prolactin	[143,152,182,185,186,187]
	Heat stress	Increased body temperature	[146,188]
Lysergic acid	Fescue toxicosis	Lysergic acid is a major breakdown compound from ergovaline in rumen	[153,189]
		1000 times less potent than ergovaline as a vasoconstrictor	[183,190]
**Indole-Diterpenoids**			
Epoxyjanthitrems	Staggers	Can be intense but short lived	[191,192]
Lolilline	Not tremorgenic		[193]
Lolitrems A, B, and F	Ryegrass staggers	Neurotoxin that affects muscular coordination; delayed onset but persistent; marked increases in respiration rate, heart rate, and blood pressure.	[84,152,158,160,193,194,195,196,197,198]
31-epi-Lolitrem B	Not tremorgenic	-	[193]
Lolitrem E	Minor tremorgen	Inhibitor of mitotic kinesin (Eg5)	[199,200]
Lolitriol	Not tremorgenic	-	[201]
Paspaline	Not tremorgenic	-	[198]
Paxilline	Moderate tremorgen	Fast acting but short longevity; marked increases in respiration rate, heart rate. and blood pressure.	[160,201,202,203,204,205]
Terpendole C	Tremorgen	Fast acting, intense but short lived	[206]
Terpendole M	Mild tremorgen	Short lived	[207]
**Pyrrolopyrazine Alkaloid**			
Peramine	No known mammalian toxicity	Possible association with causing diarrhoea, but later proven incorrect	[169,208,209]
**Pyrrolizidine Alkaloids** [175]			
N-acetyl loline (NAL)	No known mammalian toxicity	-	[168,175,210]
N-acetylnorloline (NANL)	No consistent mammalian toxicity	-	[168,174,175,210]
N-formyl loline (NFL)	No known mammalian toxicity	-	[168,175,210]

**Table 3:** The productivity and health of young sheep (30 per treatment) grazing either endophyte-free or endophyte-infected perennial ryegrass during summer and autumn periods between 1992 and 1995 on unirrigated pasture in Canterbury, New Zealand. (Taken from [165]).

Animal Trait	Endophyte-Free	Endophyte-Infected (Standard Strain)	Level of Significant Difference
Daily liveweight gain (g/head/d)	52	30	**
Ryegrass staggers score (0–5 scale)	0	3.3	**
Dags score (0–5 Scale)	0.3	2.3	**
Flystrike (% affected)	2	15	**
Rectal temperature (°C)	40.2	40.5	*
Plasma prolactin (ng/mL)	198	90	**

** *p* < 0.01; * *p* < 0.05.

### 3.2. Plant Persistence and Yield

The association between *Epichloë* endophyte presence that resulted in animal health and welfare issues led to the logical conclusion that *Epichloë* endophytes were problematic and needed to be removed from grasses. This was easily achieved because it was found that *Epichloë* strain survival in seed was negatively impacted by high temperatures and humidity [211]. The removal of *Epichloë* endophytes from sown pasture quickly led to the discovery that *Epichloë* endophytes were required for grass persistence through providing resistance/tolerance to both biotic and abiotic stresses [212,213,214,215,216]. The presence of *Epichloë* endophytes in leaf material can also increase the tolerance of grasses to herbivory [217].

### 3.3. Epichloë Effects on Abiotic Stresses

*Epichloë* endophytes have been demonstrated to improve drought tolerance in tall fescue [218,219,220,221,222,223,224,225,226,227], perennial ryegrass [228,229], and *Agrostis* [230]. However, other studies have shown no benefit of endophyte infection on drought tolerance of grasses [224,231]. It has been proposed with good evidence that interactions between plant genotype and fungal endophyte strain may explain inconsistent responses to drought due to endophyte infection [219,232,233,234,235,236,237,238,239]. Other abiotic stresses that influence plant growth and persistence that have been to some extent ameliorated by *Epichloë* endophytes include salinity [240,241,242], improved phosphorus uptake from insoluble sources [243] or nutrient poor soils [244], and tolerance to heavy metal (nickel and cadmium) stresses [245,246].

### 3.4. Epichloë Effects on Invertebrates

*Epichloë* bioactivity against insect pests were reported in the early 1980s [247]. In New Zealand, the major negative impact on ryegrass persistence is caused by a range of insect pests, some native and some introduced [248], and is often compounded by abiotic factors such as drought [249]. Ergot alkaloids, indole diterpenes (e.g., lolitrem B and epoxyjanthitrems), peramine, and the saturated aminopyrrolizidines (lolines) are alkaloids expressed by *Epichloë* strains that can protect the host plant from a range of insects [250,251] (Table 4) and can also result in anti-herbivore effects [30].

Peramine does not appear to control any pasture insect pests other than Argentine stem weevil [84,247,326].

A number of important pasture pests have to date not been shown to be controlled by specific strains or different species of *Epichloë* endophytes. These include blackheaded pasture cockchafer (*Aphodius tasmaniae*) in Australia [262,367], tobacco hornworm (*Manduca sexta*), tobacco budworm (*Heliothis virescens*), redlegged grasshoppers (*Melanoplus femurrubrum*) [368], the aphids *Sitobion avenae* [326], *Metopholophium dirhodum* and *Sitobion fragariae* [325], and the nematodes *Helicotylenchus pseudorobustus* [356], *Paratylenchus*, and *Tylenchus* [369].

### 3.5. Epichloë Effects on Other Microorganisms

*Epichloë* endophytes have frequently shown a negative impact on pathogens of grasses in planta [370,371] (Table 5). In vitro testing using dual culture assays have also often shown some antifungal effect from *Epichloë* [372,373,374,375,376], but these do not necessarily predict in planta effects [373]. Mechanisms for preventing disease in host plants by *Epichloë* may include (a) expression of volatile organic compounds to prevent insect attack which may transfer pathogens, (b) occupation of similar ecological niches in the plant, (c) enhancing the host plants growth, particularly at establishment, and/or (d) production of antifungal molecules, proteins, antioxidants, alkaloids, phytohormones, and phenolic compounds [371]. Interestingly, it has been shown that the *Epichloë* symbiosis strongly influences the endophytic fungal community (including pathogens) in the leaves of its host plant (tall fescue) so that the relative abundance of other fungal taxa can be quite different from *Epichloë* free plants [377]. However, the same study showed that there were only negligible effects of *Epichloë* on bacterial community structures in plant leaves. Rhizosphere communities are also affected by *Epichloë*, the presence of which increases species richness, particularly of Firmicutes in colonised tall fescue plants [378]. The diversity of root-associated bacterial and fungal communities was, however, found to decrease with *Epichloë gansuensis* within its host grass *Achnatherum inebrians*, but this interaction enhanced the diversity and richness of the rhizosphere soil bacterial community [379,380]. Within the phyllosphere, particular epiphytic bacterial microflora was observed to be selected for in endophyte-infected tall fescue associations [381]. Interestingly, it has been found that an increased population of plant-growth promoting bacteria in infected seed compared to endophyte-free varieties, may provide a non-direct mechanism by which *Epichloë* could possibly improve reproductive plant processes [382]. These studies demonstrate that microbial keystone species such as *Epichloë* can impact the host’s microbial community structures, which in turn can affect plant performance and ecosystem functions associated with the plant.

### 3.6. Epichloë Effects on Plant Growth

*Epichloë* presence can improve host establishment, growth, survival, tillering, and seed production [156,404]. Using clonal ryegrass genotypes, it has been shown that there can be significant improvements in yield of leaf, pseudostem, and root due to *Epichloë* endophyte infection compared with uninfected plants [405]. However, often the endophyte will interact with genotype to influence relative growth rate and productivity [406]. From a physiological viewpoint *Epichloë* endophyte in perennial ryegrass contributed to maintaining the photosynthesis mechanism under zinc stress, although it did not significantly modify net photosynthesis [407].

## 4. Delivering *Epichloë* into Managed Pastoral Systems

The impact of *Epichloë* endophytes has been of greater interest in New World pastures than in Europe driven by enhancing productivity and persistence of the host species [408]. The demonstration and realisation that *Epichloë* endophytes were important for grass persistence in these temperate pastures led to the creation of novel host plant–endophyte strain combinations that greatly enhance the persistence of the grass but with nil or much reduced (acceptable and manageable) adverse impacts on animals [6,409]. The process to deliver *Epichloë* endophytes to commerce requires a range of science capability and testing to ensure reliable bioactivity against biotic stresses that enhances plant survival while ensuring good animal health and welfare outcomes [6,410,411,412]. Through this process a number of novel *Epichloë* strains have been delivered and are now commercially used in New Zealand, USA and South America.

### 4.1. Case Study—AR1^TM^ for Ryegrass

The animal health and welfare issues created by the expression of ergovaline and lolitrem B led to the search for *Epichloë* strains that did not express these alkaloids, but were still able to provide the grass plant with resistance to major pasture pests. In New Zealand, during the 1990s, this was Argentine stem weevil and the endophyte released commercially to provide resistance while not causing ryegrass staggers was AR1 [280,413]. AR1 associations produce peramine but do not produce lolitrem B or ergovaline [414,415]. However, while effectively controlling Argentine stem weevil and pasture mealy bug, AR1 has only a moderate effect on African black beetle [282] (Table 6). AR1 can also be more susceptible to root aphid when compared to the same ryegrass germplasm without endophyte [259,416].

Released in 2001, AR1 quickly gained prominence in the market and become an endophyte of choice [12,417,418]. Over a 3-year period cows grazing AR1-infected ryegrass pastures produced 318 kg milk solids per cow per season while cows grazing standard-endophyte-infected pastures produced only 292 kg milk solids per cow, a significant 9% difference [419]. Other dairy grazing trials have demonstrated milk production increases of 6.7% [420] and up to 14% [421]. Mean summer–autumn growth rates were 170, 150, and 102 g/head/d for weaned lambs grazing cultivars with standard endophyte, nil endophyte, and AR1 endophyte, respectively [LSD_0.05_ = 48 g/head/d] [417]. These increases in production, without any endophyte associated animal health problems, have led to an unprecedented uptake of this technology by New Zealand pastoral farmers [12,422].

**Table 6:** Effects of AR1 endophyte strain in perennial ryegrass on pasture pests. (Taken from [282]).

Insect Pest	Endophyte Strain		
	Nil	Standard	AR1
**Argentine Stem Weevil**			
% tillers with larval damage	34 ^b^	4 ^a^	1 ^a^
**African Black Beetle**			
% tillers damaged by adults—6-month-old plants	52 ^c^	8 ^a^	22 ^b^
% plants damaged by larvae	58 ^b^	36 ^a,b^	28 ^a^
**Pasture Mealy Bug**			
Number per core	23 ^b^	0.6 ^a^	0 ^a^
**Root Aphid**			
Number per core	1.4 ^a^	3.5 ^a^	2.4 ^a^

^a,b,c^ Within a row, means without a common superscript letter differ significantly (*p* < 0.05).

### 4.2. Case Study—AR37^TM^ for Ryegrass

Despite the success of AR1 in controlling the impact of Argentine stem weevil on ryegrass persistence, a loss of plants began to occur through the early 2000s and this was due to the presence of other pests that were not controlled by AR1 [248,300]. Notably, these included African black beetle [423], another introduced pest and the two native pests, grass grub and porina [424]. Also impacting persistence were root aphid [259] and pasture mealy bug [271]. The AR37 endophyte was identified in the early 1990s and was shown to not produce any known problematic alkaloid compounds, but did produce a unique set of epoxyjanthitrem compounds [66,425]. These compounds have been linked to staggers in sheep, but they tend to be less frequent and less severe than those caused by lolitrem B [191,417,426]. Ryegrass staggers has not been recorded in dairy cows grazing pastures infected with AR37 endophyte [427].

In New Zealand, AR37 was found to confer a wide range of tolerance to insect pests, including Argentine stem weevil, African black beetle, root aphid, pasture mealy bug, and porina [248,259,260,261,263,271,300,352,353,416,428,429,430,431] (Table 7). The high level of resistance to the ubiquitous root aphid may be one of the factors that give plants infected with AR37 a yield advantage in nation-wide field trials [432]. AR37 also provided increased ryegrass tiller numbers, root mass and depth, persistence, and higher yields at critical times of the year [432]. With these significant benefits provided by AR37, farmers have learnt to manage the potential downside associated with epoxyjanthitrem compounds such that staggers events are rarely reported.

In New Zealand, AR37 provides significant benefits to sheep farmers through providing improved growth during the summer and autumn. During this period, lambs on pure ryegrass pastures, over a 6-year period, averaged 44 g/head/day on standard endophyte, 129 g/day on nil-endophyte and 131 g/day on AR37 infected pastures, representing increases in lamb growth of 198% over standard endophyte [417]. Total milk solids production over three consecutive lactations were not affected by use of AR37 compared with standard endophyte, indicating that AR37 is a choice of novel endophyte for pasture renewal when local insect pest populations are high [433].

In Australia, under dairy management and supplementary feeding regimes common to south-eastern Australia, the novel endophytes AR1 and AR37 had no effect on the milk production compared with the standard endophyte and did not cause ryegrass staggers [262]. They also noted that AR37 gave protection against pasture tunnel moth (*Philobota* spp.), root aphid, and an unidentified species of mealybug.

### 4.3. Case Study—Endo5^TM^ and NEA Endophytes for Ryegrass

Another approach to providing efficacious endophyte for improving ryegrass persistence was to identify *Epichloë* strains that produced little or no lolitrem B and only low levels of ergovaline. This resulted in the identification and subsequent commercialisation of the branded endophytes Endo5 (originally marketed as Endosafe) [430], NEA (which is strain NEA2) [434], NEA2 (mixture of strains NEA2 and NEA6) [435], and NEA4 (mixture of strains NEA2 and NEA3) (dxgh891opzso3.cloudfront.net › files › NEA4 booklet; [435]). The strategy behind these types of endophytes was to identify strains where ergovaline concentrations are high enough to protect against insect attack, but low enough to have minimal impact on grazing animals [436]. While NEA2 endophyte does protect ryegrass against African black beetle and pasture mealybug [248] and Argentine stem weevil [316] it does not protect ryegrass against porina or the mealybug *Phenococcus* sp. [264]. For protection against Argentine stem weevil, NEA2, which produces peramine has shown some resistance in the diploid cultivar Trojan [437], but little protection when in tetraploid cultivar Bealey [248,431,438]. Endo5 provides good protection against Argentine stem weevil, African black beetle, pasture mealybug [248], and root aphid [264], but not against grass grub [248]. This study also showed that for the NEA type endophytes, even though they express some level of ergovaline, they did not protect the host plant against root aphid.

Some of the NEA branded endophytes, such as NEA2 may also express low levels of ergovaline [434]. This however allows for the potential risk of ergovaline rising to toxic levels in some seasons or in adverse environments [439], which is predicted to occur more frequently due to climate change. It has been concluded that when ambient temperatures are suitable, NEA2-branded endophytes, just like standard endophyte, have the potential to express concentrations of ergovaline sufficient to induce heat stress in grazing sheep [434]. Others have also noted that ryegrasses infected with NEA2/3 (branded NEA4) and NEA2/6 (branded NEA2) endophytes had similar or higher concentrations of ergovaline than standard endophyte-infected ryegrass [440]. The impacts of ergovaline in New Zealand pastures has been well reviewed and found that ergovaline in standard endophyte-infected pastures can reach concentrations sufficient to cause toxicosis when ambient temperatures are suitable [439].

### 4.4. Case Study—Happe and U2 Both Fescue Epichloë Strains for Use in Ryegrass

Unlike *Epichloë* endophytes from ryegrass, those found in fescue can express lolines which are animal safe and yet have insecticidal properties against a range of insect pests (Table 4). Moving *Epichloë* endophytes from fescues into ryegrass through isolation and inoculation has been attempted but has proven challenging. Only two have moved to commercialisation, Happe, a unique endophyte of the species *E. siegelii* [36], and U2 (*E. uncinatum*) [302,303,441], both from meadow fescue.

Perennial ryegrass inoculated with Happe have shown reasonably high expression of loline alkaloids [172], which may be sufficient to give protection against major insect pests including the grass grub.

U2 has been inoculated into festulolium hybrids [442] in an attempt to improve seed transmission rates. The principle loline type expressed by U2 in festulolium hybrids was NFL (68% of total lolines), followed by NAL (23%), and NANL (8%) [443]. The endophyte strain U2 has shown to provide good resistance against a range of insect pests, including grass grub [92,321], African black beetle [302], Argentine stem weevil [431,444], and crickets [302].

### 4.5. Case Study—AR542 and AR548 (MaxQ^TM^, MaxQII^TM^, and MaxP^TM^) for Tall Fescue

Fescue toxicosis has been associated with the presence of high ergovaline expressing *Epichloë* strains in tall fescue [161,445,446]. Replacement with endophyte strains that do not produce ergovaline has been successfully achieved and led to the release of strain AR542 (MaxQ) in the USA in 2000 [447,448,449,450,451]. This was later replaced with AR584 (MaxQII), a strain that provided all the benefits of AR542 but had improved seed borne transmission and storage characteristics [452]. AR542 expresses peramine and the loline compound NANL, while AR584 expresses peramine and the three loline compounds NFL, NAL, and NANL [284].

The MaxQ brand of endophytes has provided agronomically superior tall fescue cultivars that do not cause any fescue toxicosis symptoms [439] and has been described as a “win-win” outcome [411]. In New Zealand, MaxP^TM^ endophyte reduces damage by African black beetle, Argentine stem weevil, pasture mealy bug, grass grub, and root aphid in a range of tall fescue cultivars [209,267,284,300,453]. Other insect pests that these ergot alkaloid free endophytes control include fall armyworm [454], corn flea beetle (*Chaetocnema pulicaria*) [455], and bird cherry oat aphids [341,456]. Sheep show no difference in preference to grazing MaxP^TM^ endophyte containing tall fescue compared with nil-endophyte tall fescue [457]. Lambs grazing MaxQII^TM^ containing tall fescue gained an average of >139 g d^−1^, more than twice the 68 g d^−1^ gained by animals grazing endophyte-infected Kentucky-31 [458].

Brood-balls from the dung beetle *Onthophagu taurus* preferred dung from cows grazing tall fescue Texoma MaxQ II while dung from cows grazing tall fescue Kentucky31 and BarOptima PLUSE34 were avoided [459]. Both *O. taurus* and the other beetle species *Digitonthophagus gazella* preferred dung from Texoma MaxQII compared with endophyte-infected Kentucky31 pasture.

### 4.6. Case Study—E34 for Tall Fescue

E34 (also known as BE9301A) produces ergovaline but at lower levels (<10% to 50% depending on host germplasm and environment) than standard endophyte Kentucky 31 tall fescue, resulting in a significantly higher average daily gains of steers of 1.93 lb compared with 1.29 lb, respectively [460]. In field trials over two years in two USA states the value of novel endophyte varieties that produce no ergot alkaloids was confirmed, and it was demonstrated that while varieties such as BarOptima Plus E34 express consistently lesser levels of ergot alkaloids than Kentucky 31 [461] (Table 8), they can elevate in some circumstances to levels that are greater than that considered safe for livestock based on previous studies [152,462].

Comparison of BarOptima and MaxQ (AR542) tall fescue endophytes, however, does show that animal performance in terms of average daily weight gain of cattle of both was similar to endophyte free tall fescue and considerably better than on the endophyte-infected Kentucky 31 pasture (Table 9). Grazing days on endophyte free pasture was low due to poor pasture resilience without the endophyte. Interestingly, blood serum prolactin levels were slightly lower for BarOptima than endophyte free and MaxQ (Table 9).

### 4.7. Case Study—Protek (E647) for Tall Fescue

Protek is an endophyte that does not produce ergovaline or any other ergopeptide alkaloids and in combination with tall fescue increased yields of young seedlings by 20 to 100% and increased resistance to African black beetle, which reduces severely damaged tillers of seedlings by 20% to 45% depending on host germplasm [464]. Average daily weight gain of ewes grazing over three years showed that ewes on Kentucky 31 achieved only 32 mg/head/day while those on tall fescue cultivar Martin E647 achieved 102 mg/head/day which compared favourably with a nil-endophyte Martin which achieved 103 mg/head/day [464].

### 4.8. Case Study—ArkShield in Tall Fescue

Also known as Strain 4 or ArkPlus, ArkShield is a strain that does not produce ergot alkaloids but does produce the lolines compounds NFL and NAL at about 50% and 100% of the levels expressed in endophyte-infected Kentucky 31 [465] (Table 10). Compared with Kentucky 31, ArkShield improved animal live weight gains and increased blood serum prolactin levels (Table 10).

### 4.9. Delivery of Commercial Novel Epichloë Endophytes

Effective delivery of these novel endophyte infected cultivars requires care with management of seed crops ensuring appropriate fungicides are used and seed moistures levels are 10% to 12% at seed harvest [209]. When processed the seed must be packaged appropriately and stored at low temperature and humidity until ready to be sown. Quality control systems and monitoring of endophyte viability is required through the retail and distribution chain [130,466,467]. This has been agreed among suppliers of *Epichloë* endophyte products.

Endophyte viability in seed should be above 70% at the point of sale to ensure farmers are purchasing a quality product [468,469]. Ensuring that the supply chain from science through seed companies and retailers to the end-user farmer are well resourced and consistent is crucial in the uptake and use of endophyte technologies in pastoral agriculture [422,470,471]. This requires using well designed production and quality assurance guidelines to deliver a high-quality endophytic seed technology, giving the farmer confidence that it will provide the promised benefits [466].

## 5. Future Opportunities

A significant challenge for delivering future *Epichloë* stains of commercial value for tall fescue and ryegrass is the scarcity of new and novel variation available in natural strains. Considerations to overcome this might include:

- Genetic modification of *Epichloë* using traditional gene insertion or deletion [472,473] and the more recent CRISPR (clustered regularly interspaced short palindromic repeats)-Cas9 (CRISPR-related nuclease 9) system [474] to either:
  ➢ manipulate existing alkaloid pathways to increase the expression of mammalian safe intermediate pathway compounds, whilst removing toxic end products;
  ➢ insert secondary metabolite genes to make new compounds in planta; and/or
  ➢ repair non-functional genes (pseudogenes) in secondary metabolite pathways to restore lost bioactivity
- Using DNA marker information to improve the efficiency of selection for endophyte compatibility in host plants when moving strains across taxa [475];
- Identify and determine the function of bacteria associated with *Epichloë* in planta [476]; and
- Develop an understanding of molecular processes that underpin compatibility between the host and fungal endophyte so that movement of *Epichloë* strains across widely separated taxa can be achieved successfully, ensuring normal phenotypes and good transmission through seed [475,477,478]. This may require genetic manipulation of genes in both partners to be successful, but on the other hand, the genetic information may simply be used to screen for compatible endophyte and host germplasm that are more likely able to form stable and beneficial symbioses.

*Epichloë* endophytes are known to produce a large number of secondary metabolites, many in planta [67,479], but some at low amounts in culture [83]. Exploitation of these has not as yet been realised but may result in bioactives that have anthelmintic effects, impacts on methanogenic microbes in ruminants, and pesticidal [480] and antifungal effects [374,401,481,482].

## 6. Concluding Comment

*Epichloë* endophytes have been found in a wide range of wild grasses across most temperate regions of the world. Strains of *Epichloë* are characterised by the range of alkaloids they are capable of producing in planta. These can provide an adaptive advantage to the host grass through reducing herbivory of ruminants, providing resistance to some pests and pathogens, and improving tolerances to some abiotic stresses. In some temperate regions, namely New Zealand, Australia, and USA, it has been demonstrated that ryegrass and tall fescue pastures require plants to be infected with *Epichloë* for them to yield well and persist. However, for *Epichloë* strains to be effectively commercialised, their characterisation is required to ensure that the expression of specific alkaloids while providing an advantage to the plant do not also result in animal health and welfare concerns. This has been achieved, with several different *Epichloë* strains being successfully commercialised and widely used by pastoral farmers.

## Figures and Tables

**Table 4:** Invertebrate organisms (insects, nematodes and molluscs) impacted by *Epichloë* endophytes; for other older references related to effects of *Epichloë* endophytes in ryegrass and tall fescue on insects, refer to Breen (1994) [252].

Organism	Impact	Alkaloid Involved	*Epichloë* Strain/Type	Reference
**Insects**				
*Acheta domesticus*—house crickets	Toxic to nymphs	ns *	Ryegrass types	[253]
*Adoryphorus coulonii*—Red-headed cockchafer	Reduced (10–20%) root consumption at >1000 µg/g DM	Loline	Meadow fescue types	[254]
*Agallica constricta—*leaf hopper	Resistance	ns	Fescue types	[255]
*Agrostis ipsilon*—Black cutworm	Deterrence and toxicity	Ergovaline and/or ergine most potent, with lolines also effective	*E. lolii* x *E. typhina* hybrid from ryegrass	[256,257]
*Aploneura lentisci*—root aphid	Reduced survival; possible neurotoxin	Unknown (in case of AR5), and possibly epoxy janthitrems	AR37, AR5, AR6, and standard ryegrass endophyte	[258,259,260,261,262,263,264]
	Reduced root aphid numbers per plant	Possibly lolines—NFL and NAL	Fescue types	[265,266,267]
	Minimal effect	Despite having similar ergovaline levels in roots as AR5	NEA2 and NEA6 endophytes	[264]
	Increased numbers	ns	AR1 endophyte	[268]
*Balanococcus poae*—Pasture mealybug	Reduced survival	ns	Ryegrass types including AR1	[258,269,270,271]
	Reduced infestation	ns	Fescue types that do not express ergovaline	[272]
*Blissus leucopterus hirtus*—hairy chinch bug	Deterrence and toxicity to larvae and adults	ns	Fescue and ryegrass types	[273,274,275,276,277]
	No effect		Fescue types	[278]
*Costelytra zealandica* or *C. giveni—*Grass grub	Reduced root feeding and larval weight gain; a deterrent effect	Loline and increased levels due to grass grub attack	Fescue and meadow fescue types; *E. uncinatum*	[91,92,279,280,281,282,283,284,285,286]
*Cerodontha australis—*wheat sheath miner	Toxicity or deterrence to larvae, but no effect on oviposition	ns	AR47 and AR48 ryegrass strains	[287]
*Crambus roman*—sod webworm	Deterrent	ns	Ryegrass types (turf)	[288]
*Ctenocephalides felis*—cat flea larvae	Contact toxicity	NFL	Fescue types	[289]
*Cyclocephala lurida*—southern masked chafer	Reduced numbers	ns	Fescue types	[217]
*Diuraphis noxia—*Russian wheat aphid	Toxic to nymphs and adults; deterrent to adults	ns	Ryegrass and fescue types	[290,291]
*Draeculacephala* spp.—leaf hopper	Resistance	ns	Fescue types	[25,292]
*Drosophila melanogaster*—fruit fly	Toxic to adults	ns	Fescue types	[293]
*Exitianus exitiosus—*leaf hopper	Resistance	ns	Fescue types	[255]
*Exomala orientalis*	Reduced survival	ns	Fescue types	[294]
*Graminella nigrifrons—*leaf hopper	Resistance	ns	Fescue types	[255]
*Graphania mutans*—cutworm	Not a deterrent, but disrupted development	Peramine	Ryegrass types	[295]
*Heteronychus arator*—African black beetle	Antifeeding effect on adults	Ergopeptine alkaloids - ergotamine, ergovaline, ergocryptine	Standard ryegrass endophyte; AR22, AR12 endophytes	[260,270,280,296,297,298,299,300]
	Reduced numbers	ns	AR37 endophyte	[260]
	Deterrent, antifeeding effect on larval and adult stages	Loline	Fescue and meadow fescue types; *E. uncinata*	[254,301,302]
	No effect	Peramine, lolitrem B, paxilline, festuclavine, lysergol, and lysergic acid amide	Ryegrass and fescue types	[280,297,298]
*Lepidogryllus* spp.—mottled field cricket	Deterrent	Loline	Meadow fescue types; *E. uncinatum*	[303]
*Listronotis bonariensis*—Argentine stem weevil	Feeding deterrent for both adults and larvae; reduced oviposition	Peramine—higher concentration required to control larvae	Ryegrass types; AR1, AR5, NEA2 endophytes	[84,245,260,270,299,304,305,306,307,308,309,310,311,312,313,314,315,316,317]
	Feeding deterrent and toxin of larvae, but not adults	Lolitrem B	Ryegrass types	[315,318,319,320]
	Feeding deterrent	Paxilline	Ryegrass types	[84]
	Reduce larval damage of tillers	ns	AR37 endophyte	[260]
	Feeding deterrent and death of larvae	Loline level above 400 µg/g DM; NANL possibly more potent than NFL at moderate concentrations	Meadow fescue types	[279,321,322,323]
	Feeding deterrent	Ergovaline; ergocryptine; ergotamine	Ryegrass types	[295,324]
	No effect		Ryegrass and fescue types	[325]
*Oncopeltus fasciatus*—large milkweed bug	Feeding deterrent and toxic	NFL	Fescue types	[140,326]
*Ostrinia nubilalis*—European corn borer larvae	Toxic effects and reduced larval weight gain	NAL	Fescue types	[327]
*Parapediasa teterella—*bluegrass webworm	Deterrent, reduced feeding	ns	Fescue and ryegrass types	[328,329,330]
*Periplaneta Americana*—American cockroach	Contact toxicity	NFL	Fescue types	[289]
*Phenococcus solani—*mealybug	Reduced numbers	ns	Fescue types	[331]
*Philobota* spp.—Pasture tunnel moths	Reduced numbers	ns	AR37	[262]
*Popillia japonica*—Japanese beetle larvae	Contact toxicity	NFL	Fescue types	[289]
	Reduced feeding	Particularly NFL and NAL; and lesser effect of ergotamine, ergonovine, ergocryptine	Fescue types	[294,332]
	Inconsistent effects		Fescue types	[294,333]
	No effect		Fescue and ryegrass types	[334,335,336,337]
*Prosapia bicincta—*leaf hopper	Resistance	ns	Fescue types	[255]
*Pseudococcidae*—mealybugs	Reduced numbers	ns	AR37	[262]
*Rhopalosiphum padi—*aphid	Feeding deterrent and toxic	Loline	Fescue types	[325,326,338,339,340,341]
	Reduced numbers	ns	*E. gansuense*	[342]
	No effect	Ergovaline	Ryegrass and fescue types	[338]
*Rhopalosiphum maidis*—Corn leaf aphid	Some resistance, but less than for *R. padi* and *S. graminum*	ns	Ryegrass types; lesser impact of fescue types	[326]
*Schizaphis graminum*—aphid	Toxic causing reduced numbers	Loline	Fescue types; *E festucae* and *E. uncinatum*	[326,327,340]
	Feeding deterrent and toxic	Peramine	Ryegrass and fescue types	[338]
	No effect	Ergovaline		
	Resistance	ns	Fescue types	[140]
*Sphenphorus parvulus*—Bluegrass billbug	Resistance/ toxicity to adults	ns	Ryegrass and fescue types (turf)	[288,292,343,344]
*Spodoptero frugiperda*—fall army worm	Reduced worm survival and liveweight gains	ns	Fescue and ryegrass types	[345,346,347,348]
		NFL, NAL	Fescue types	[327]
		Ergotamine, ergonovine, ergocryptine	Fescue types	[349]
*Spodoptera eridania—s*outhern army worm	Toxic	ns	Ryegrass types	[350]
*Teleogryllus commodus*—black field cricket	Deterrent	Loline	Meadow fescue types; *E. uncinatum*	[303]
*Trigonotylus caelestialium*—rice leaf bug	Resistance	Loline	Fescue types	[351]
*Wiseana cervinata—*Porina	Reduced survival	ns	AR37 ryegrass type	[80,192,256,352,353]
	Reduce feeding and weight gain	Paxilline		[354]
		Loline	Fescue types	[282]
**Mites**				
*Tetranychus cinnabarinus*	Reduced numbers	ns	*E. gansuense*	[342]
**Nematodes** (refer to [355] Cook and Lewis 2001)				
*Helicotylenchus pseudorobustus—*spiral nematodes	Reduced numbers	ns	Fescue types	[356]
*Meloidogyne marylandi*	Fewer egg masses and eggs and reduced infection	ns	Fescue types	[356,357,358]
	Reduced infection	ns, but not ergovaline	Ryegrass types	[90]
*Meloidogyne nassi*	Reduced galls and females	ns	Ryegrass types	[359]
*Paratrichodorus minor—*stubby root nematodes	Reduced numbers	ns	Fescue types	[360]
*Pratylenchus scribneri*—Lesion nematode	Repellent and death	NFL at high concentrations; and ergovaline	Fescue types	[356,361]
	Reduced numbers	ns	Fescue types	[362,363]
	Attractant and causes death	Ergovaline, ergotamine	Fescue types	[361]
	Repellent	Ergocryptine, ergonovine	Fescue types	
	Attractant at <20 µg/m and repellent at high concentrations	NFL	Fescue types	
*Pratylenchus* spp.	Reduced numbers in soil	ns	Ryegrass types	[364,365]
*Tylenchorhynchus acutus—*stunt nematodes	Reduced numbers in soil	ns	Fescue types	[362]
**Molluscs**				
*Deroceras reticulatum*	Reduced feeding	Lolitrem B and possibly lolines	Used artificial diets incorporating the secondary metabolites	[366]
	No effect	Peramine		
	Stimulated feeding	Ergotamine and ergovaline		
	Attractant	Paxilline, lolitriol, a-paxitriol and b-paxitriol		

* ns = not specified.

**Table 5:** Pathogens impacted by *Epichloë* endophytes in planta.

Pathogen	Impact of Endophyte	Alkaloid Involved	*Epichloë* Strain/Type	Reference
*Alternaria alternata*	Moderate resistance	Enhanced superoxide dismutase or peroxidases activity	Ryegrass types	[383]
	Reduced incidence of infection	ns *	Host: *Elymus cylindricus*	[384]
*Bipolaris sorokiniana*	No effect in planta		*E. bromicola*	[375]
	No effect in planta		*E. gansuensis*	[342]
	Reduced incidence of infection	ns	Host: *Leymus chinensis*	[385]
		ns	Fescue types	[386]
	Resistance to infection	Enhanced superoxide dismutase or peroxidases activity	Ryegrass types	[383]
*Blumeria graminis*—powdery mildew	Lower disease incidence	ns	*E. gansuensis*	[342,387]
*Cladosporium* sp.	No effect in planta		*E. bromicola*	[375]
*Claviceps purpurea*	Reduced infection unless plants water stressed	ns	Annual ryegrass types	[388]
*Cochliobolus sativus*—soil pathogen	No effect		Fescue types	[389]
*Curvularia lunata*	No effect in planta		*E. bromicola*	[375]
	Moderate resistance	Enhanced superoxide dismutase or peroxidases activity	Ryegrass types	[383]
	Reduced incidence of infection	ns	Host: *Leymus chinensis*	[385]
	Reduced disease symptoms	ns	Fescue types	[390]
*Drechsler sp*.	Reduced incidence infection	ns	Fescue types	[386]
*Drechslera erythrospila*	Inhibited hyphal growth	ns	Ryegrass and fescue types	[373]
	Reduced disease symptoms in planta	Protease and endoglucanase activity	*E. fesctucae*	[374]
*Drechslera siccans*—brown blight	Resistance to infection	ns	Ryegrass types	[370]
*Fusarium avenaceum*	Resistance to infection	Enhanced superoxide dismutase or peroxidases activity	Ryegrass types	[383]
*F. avenaceum*	Reduced incidence of infection	ns	Host: *Elymus cylindricus*	[384]
*F. culmorum*	Reduced incidence of infection	ns	Host: *Elymus cylindricus*	
*F. oxysporum*	Reduced incidence of infection	ns	Host: *Elymus cylindricus*	
	Increased resistance	ns	*Fescue arizonica* type	[391]
*F. poae*	Reduced incidence of infection	ns	Fescue types	[386]
*Fusarium* spp.	No effect		Ryegrass and fescue types	[392]
	Resistance to infection	ns	Ryegrass types	[370]
*Laetisaria fuciformis*—red thread	Lower disease incidence and severity	ns	Meadow fescue types	[393]
*Microdochim bolleyi*	No effect		Ryegrass and fescue types	[392]
*Phaeosphaeria—*leaf spot	No effect		Meadow fescue types	[394]
*Puccinia graminis* subsp. *graminicola*	No effect		Fescue types	[395]
*Puccinia* spp.	No effect		*E. uncinatum*	[396]
*Pyrenophora semeniperda—*leaf spot	Reduced disease symptoms in planta	ns	Ryegrass types	[397]
Rhizoctonia blight	No effect		Fescue types	[398]
*Rhizoctonia zeae*	Reduced disease symptoms in planta	Phenolic compounds	Fescue types	[399]
	Reduced hyphal growth	ns	*E. uncinatum*	[373]
*R. solani*	Reduced incidence of infection	ns	Fescue types	[386]
*Sclerotinia homoeocarpa—*Dollar spot disease	Lower disease incidence and severity	Antifungal protein	Meadow fescue types	[400,401]
*Typhula ishikariensis*—snow mold	Increased susceptibility	ns	Meadow fescue types	[402]
*Ustilago bullata*—head smut	Suppressed infection	ns	*E. tembladerae*	[403]

* ns = not specified.

**Table 7:** The effect of AR37 endophyte strain in perennial ryegrass on insect pests. (Taken from [260]).

Endophyte Strain	Tillers Damaged by ASW (%)	Number of Black Beetles per m^2^	Tillers Damaged by Porina Larvae (%)	Number of Root Aphids per Plant *
AR37	2.1	23	13.6	2 (0.5)
Standard	2.8	17	28.7	171 (1.23)
Nil endophyte	25.7	64	34.9	244 (1.93)
LSD_0.05_	14.2	26	19.9	(0.67)

* Log-transformed data in parentheses.

**Table 8:** Mean total ergot alkaloids and ergovaline concentrations (µg kg^−1^) in the leaf blade and leaf sheath BarOptima Plus E34, and Kentucky 31 varieties of tall fescue sampled during 2012 and 2014 across Georgia and Kentucky. (Taken from [461]).

Tall Fescue Variety	Total Ergot Alkaloid Concentration (µg kg^−1^)		Ergovaline Concentration (µg kg^−1^)	
	Leaf Blade	Leaf Sheath	Leaf Blade	Leaf Sheath
BarOptima Plus E34	133 ^b^	337 ^b^	37 ^b^	343 ^b^
KY31	1667 ^a^	6312 ^a^	268 ^a^	2848 ^a^
*p*-value	<0.0001	<0.0001	<0.0001	<0.0001

^a,b^ Within a column, means without a common superscript letter differ significantly (*p* < 0.05).

**Table 9:** Mean over two years average daily gain (ADG), grazing days per ha, and blood serum prolactin levels (in February) of 11 month old calves grazed on different endophytic tall fescue pastures in the Coastal Plain region of southwestern Arkansas. (Taken from [463]).

Tall Fescue and Endophyte	ADG (kg/day)	Grazing Days per ha	Blood Serum Prolactin (ng/mL)
KY31	0.58	529	1.5
Endophyte free	1.08	384	62
BarOptima E34	0.93	553	38
Jesup AR542 (MaxQ)	0.88	611	79
SEM *	0.08	30	14

* SEM—standard error of the mean; for Jesup AR542, *n* = 2; for KY-31, EF, and BarOptima E-34, *n* = 3.

**Table 10:** Mean concentrations (µg/g of DM) in the herbage of measured ergot alkaloids and loline levels (N-formyl loline (NFL) and N-acetyl loline (NAL)), average daily weight gain (ADG) of 2 year old steers, and blood serum prolactin levels across two sites in the USA. (Taken from [465]).

Tall Fescue and Endophyte	Endophyte Infection Rate (% Viable in Seed)	Alkaloid Levels (µg/g of DM)			ADG (kg/day)	Prolactin (ng/mL)
		Total Ergot Alkaloids	NFL	NAL		
HiMag—ArkShield	94	0	161	117	0.6 ^a^	155 ^a^
KY31	80	0.70	305	117	0.34 ^b^	17 ^b^
HiMag—Nil endophyte	0	0	0	0	0.62 ^a^	108 ^a^

^a,b^ Within a column, means without a common superscript letter differ significantly (*p* < 0.05).
